# Non-Aneurysmal Subarachnoid Hemorrhage in a Patient With Type 1 Diabetes Mellitus: A Case Report

**DOI:** 10.7759/cureus.112750

**Published:** 2026-07-15

**Authors:** Dawit Y Barkesa, Gemechu F Gosse, Gutu D Kumsa, Gemechu G Beriso, Miresa K Eticha

**Affiliations:** 1 Internal Medicine, Negele Arsi General Hospital and Medical College, Negele Arsi, ETH; 2 Radiology, Negele Arsi General Hospital and Medical College, Negele Arsi, ETH

**Keywords:** ct angiography, diabetes mellitus, ethiopia, microangiopathy, non-aneurysmal subarachnoid hemorrhage

## Abstract

Non-aneurysmal subarachnoid hemorrhage (NASAH) is defined as bleeding into the subarachnoid space without an identifiable aneurysm. In patients with long-standing diabetes mellitus (DM), chronic microvascular damage may contribute to vascular fragility and hemorrhage. Emerging evidence suggests a possible association between NASAH and diabetes, particularly through microangiopathic mechanisms. We report the case of a 40-year-old female patient with a 14-year history of poorly controlled Type 1 DM (T1DM) who presented with sudden-onset severe occipital headache, neck stiffness, and vomiting. On examination, she was neurologically intact with a Glasgow Coma Scale score of 15/15, with mild nuchal rigidity. Non-contrast head CT revealed diffuse subarachnoid hemorrhage. CT angiography showed no aneurysm or vascular malformation, confirming NASAH. She was managed conservatively with nimodipine, analgesics, and supportive care, with good clinical recovery and no residual deficits. This case highlights NASAH as a potential microvascular complication of long-standing T1DM. Early recognition and appropriate imaging are essential for diagnosis, particularly in resource-limited settings, and can lead to favourable outcomes.

## Introduction

Subarachnoid hemorrhage (SAH) is a neurological emergency characterized by bleeding into the subarachnoid space, most commonly due to rupture of intracranial aneurysms [1]. However, approximately 10-15% of spontaneous SAH cases occur without an identifiable aneurysm and are classified as non-aneurysmal subarachnoid hemorrhage (NASAH) [1]. Compared to aneurysmal SAH, NASAH is generally associated with a more favorable clinical course and lower rates of morbidity and mortality.

Diabetes mellitus (DM) is a well-established risk factor for cerebrovascular disease, primarily through its effects on the macrovascular and microvascular systems [2]. While several studies have suggested that DM is not strongly associated with aneurysmal SAH [2], emerging evidence indicates a higher prevalence of DM among patients with NASAH [3,4]. This observation has led to the hypothesis that diabetic microangiopathy, endothelial dysfunction, and increased vascular fragility may contribute to the pathogenesis of NASAH. However, the available evidence remains limited, and no causal relationship has been established. Therefore, the proposed role of diabetic microvascular disease in NASAH should be considered hypothetical and warrants further investigation.

Here, we report a case of NASAH in a patient with long-standing, poorly controlled Type 1 DM (T1DM), in whom no conventional risk factors or vascular abnormalities were identified. This case adds to the limited literature describing NASAH in patients with long-standing T1DM and explores the potential association between chronic diabetic microvascular disease and NASAH. By documenting this rare presentation in a resource-limited setting, our report underscores the importance of considering NASAH in diabetic patients presenting with acute severe headache, while recognizing that the proposed underlying mechanism remains hypothetical.

## Case presentation

A 40-year-old female patient with a 14-year history of poorly controlled T1DM, managed with mixed insulin 70/30 at doses of 30 units in the morning and 22 units in the evening, presented to the emergency department with a sudden, severe occipital headache of one hour duration. The headache was described as the worst in her life and was associated with a single episode of non-bilious vomiting and neck stiffness. She denied trauma, loss of consciousness, seizures, or focal neurological deficits. She had no history of hypertension, smoking, alcohol use, coagulopathy, or oral contraceptive use. There was no family history of cerebrovascular disease.

On examination, the patient was acutely ill, appearing in pain, but her vital signs were within normal limits. Chest auscultation revealed clear lung fields, and heart sounds were normal without murmurs or added sounds. Abdominal examination showed a soft, non-tender abdomen with no signs of organomegaly or fluid collection.

Neurological examination revealed a Glasgow Coma Scale (GCS) score of 15/15. The patient was alert and oriented to time, place, and person. Pupils were midsized and reactive to light bilaterally. All cranial nerves were intact. Motor examination showed 5/5 muscle strength in all four extremities, with normal tone and reflexes. Sensory examination and coordination were unremarkable. Mild nuchal rigidity was observed on neck examination, raising concern for meningeal irritation; however, no other meningeal signs were identified.

Diagnosis and investigation

Laboratory investigations were largely within normal limits, including complete blood count, renal and liver function tests, and serum electrolytes. Glycemic control was poor, with a glycated hemoglobin (HbA1c) of 10.8%. Viral markers for hepatitis B and C were negative. Serologic tests, including human immunodeficiency virus (HIV), antinuclear antibodies (ANA), and Venereal Disease Research Laboratory (VDRL), were non-reactive. Detailed laboratory results with corresponding reference ranges are presented in Table 1. 

**Table 1:** Laboratory investigations with corresponding reference values HIV: human immunodeficiency virus; VDRL: Venereal Disease Research Laboratory; HbA1c: glycated hemoglobin

Parameter	Result	Reference Range	Unit
Complete Blood Count			
White Blood Count	10.8	M: 4.5-11.5, F: 4.5-11.5	×10³/µL
Lymphocytes %	32.6	20–35%	%
Neutrophils %	61.1	M: 40-75, F: 40-75	%
Hemoglobin	13	M: 13.0-17.0, F: 12.0-16.0	g/dL
Hematocrit	39	M: 41.0-54.0, F: 41.0-54.0	%
Platelets	285	150-450	×10³/µL
HbA1c	10.8	4.0–5.6	%
Renal Function Test			
Creatinine	0.67	0.5–1.1	mg/dL
Urea	18	10-50	mg/dL
Liver Function Test			
Aspartate Aminotransferase	22	10-40	U/L
Alanine Aminotransferase	25	7-54	U/L
Alkaline Phosphatase	90	44-147	U/L
Bilirubin, total	0.8	0.1-1.2	mg/dL
Bilirubin, Direct	0.2	0.0-0.3	mg/dL
Albumin	4.0	3.5-5.0	g/dL
Serum Electrolytes			
Sodium	135.9	135–145	mmol/L
Potassium	3.66	3.5–5.0	mmol/L
Chloride	98	95–115	mmol/L
pH	7.4	7.35–7.45	-
Calcium (ionized)	1.17	1.15–1.33	mmol/L
Calcium (total)	2.1	2.1–2.6	mmol/L
Serology			
Hepatitis B Surface Antigen	Negative	Negative	
Hepatitis C Virus Antibody	Negative	Negative	
HIV test	Non-reactive	Non-reactive	
Antinuclear Antibodies	Non-reactive	Non-reactive	
VDRL	Non-reactive	Non-reactive	

Funduscopic examination showed no evidence of diabetic retinopathy. Serum creatinine was within the normal range. However, urine albumin testing and formal evaluation for diabetic peripheral neuropathy were not performed during hospitalization.

**Figure 1:**
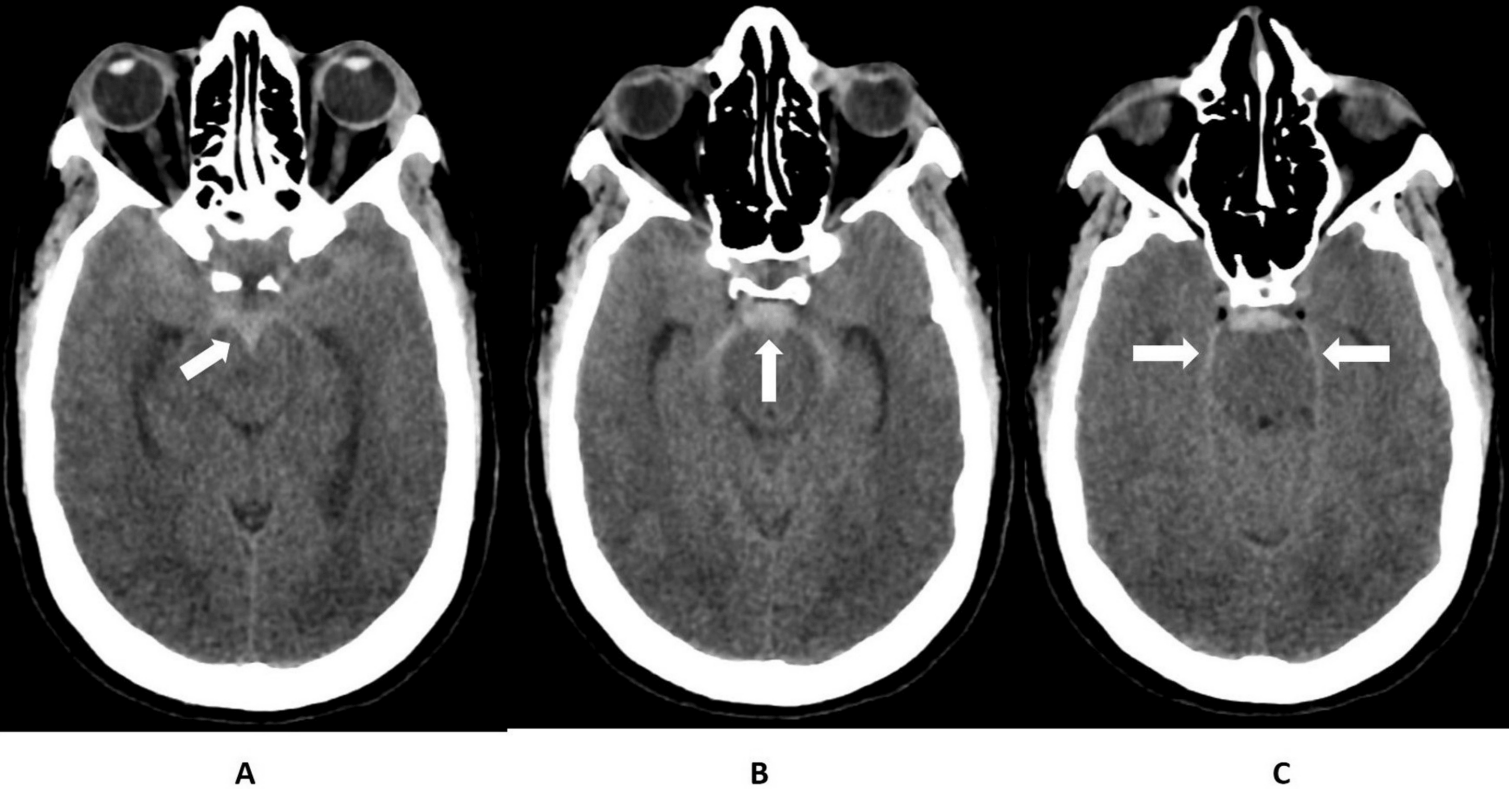
Non-contrast head CT scan showing hyper-densities along basal cisterns with a perimesencephalic hemorrhage pattern (white arrows)

**Figure 2:**
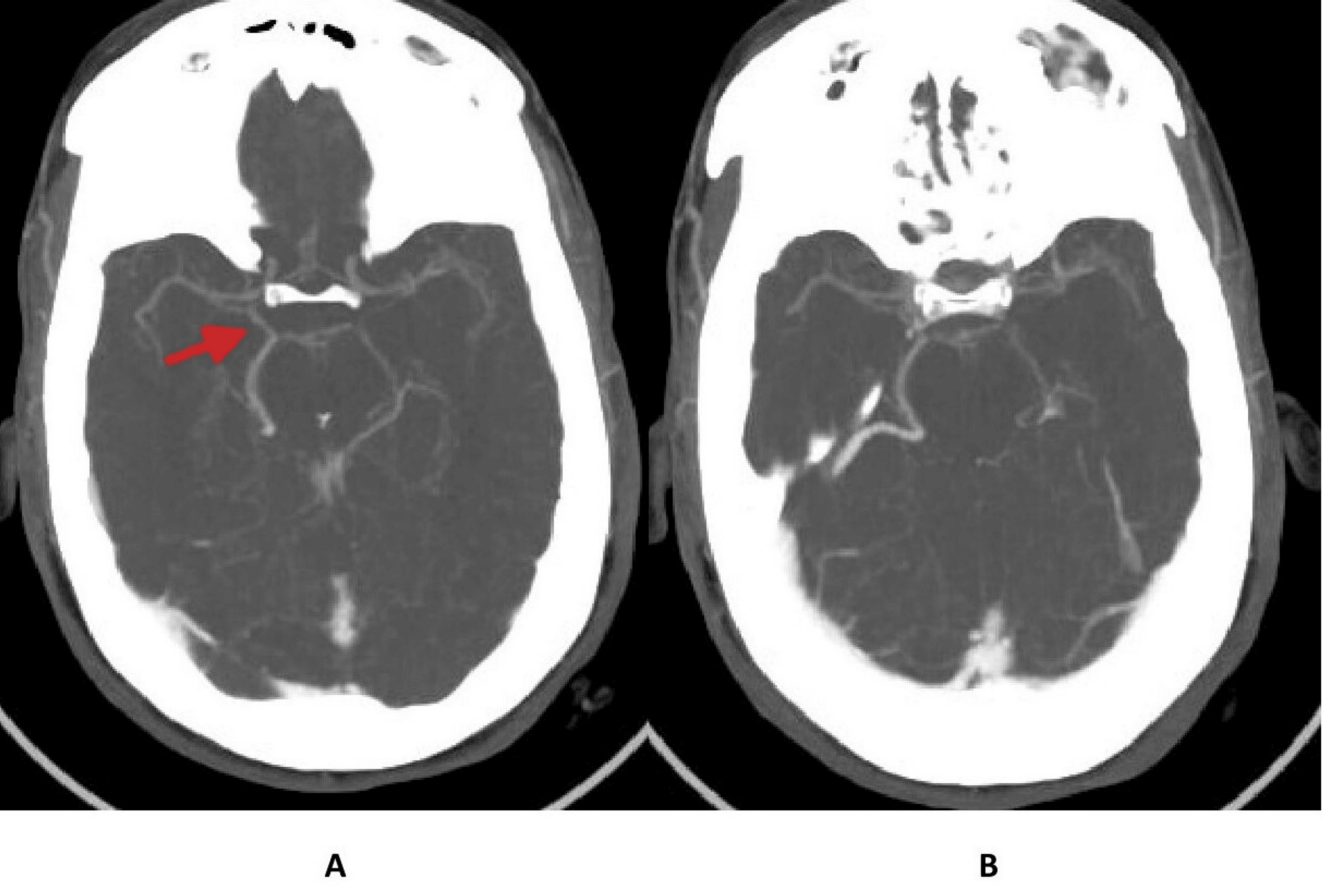
CT angiography showing acute sub-arachnoid hemorrhage with no evidence of aneurysm or vascular malformation. The circle of Willis and major intracranial arteries have normal contrast distribution without filling defects or aneurysmal dilation. There is minimal prepontine space pooling with slight residual right cavernous extension, consistent with a perimesencephalic pattern of subarachnoid hemorrhage (red arrow).

A non-contrast head CT scan was performed, revealing diffuse subarachnoid hemorrhage predominantly in the basal cisterns, with diffuse hypodensity suggestive of brain edema, but no signs of increased intracranial pressure (Figure 1). CT angiography did not demonstrate any aneurysms, arteriovenous malformations, or vascular anomalies (Figure 2), leading to the diagnosis of NASAH. Digital subtraction angiography (DSA) was not performed because it was unavailable at our institution. Grading scores were consistent with a favorable clinical presentation: Hunt and Hess Grade II [5], World Federation of Neurosurgical Societies (WFNS) Grade I [6], and Fisher Group 2 [7].

Treatment and outcome

The patient was admitted to the neurology ward for close monitoring and medical management. She was placed on strict bed rest with head elevation, and nimodipine 60 mg orally every four hours was initiated to prevent vasospasm. Anticonvulsant prophylaxis with phenytoin (100 mg orally three times daily) was given. Additional supportive management, including paracetamol, morphine, bisacodyl, and ondansetron, was provided. 

Over the course of her hospitalization, the patient remained hemodynamically stable with no new neurological deficits, apart from persistent headache and intermittent vomiting. Blood glucose levels were monitored regularly and managed using a modified sliding scale in conjunction with her baseline insulin regimen. Blood pressure and fluid balance (input and output) were closely monitored to prevent hypovolemia. Serum electrolytes, particularly sodium, were also followed carefully to avoid hyponatremia.

Given the absence of vascular anomalies and a history of poorly controlled T1DM, it was concluded that the subarachnoid hemorrhage likely resulted from microvascular fragility secondary to chronic diabetic vasculopathy. The patient was counseled on the importance of optimal glycemic control and regular follow-up to monitor for other potential microvascular complications. She was discharged after 10 days of hospitalization on oral nimodipine to complete a 21-day course, along with a revised insulin regimen (mixed insulin 70/30 at doses of 38 units in the morning and 26 in the evening, with plans for outpatient follow-up in neurology and endocrinology.

## Discussion

SAH is most commonly caused by rupture of intracranial aneurysms and is typically considered a manifestation of macrovascular disease [2]. However, NASAH represents a distinct clinical entity characterized by hemorrhage in the absence of identifiable vascular abnormalities on imaging [8]. NASAH accounts for 0.3-0.5 cases per 100,000 persons [1]. Although less common, NASAH generally follows a more benign clinical course and is associated with lower morbidity and mortality compared to aneurysmal SAH [3,8,9].

The relationship between DM and SAH has traditionally been considered weak or even protective, particularly in aneurysmal SAH [9]. However, emerging evidence suggests that this relationship may differ in cases of NASAH. Several studies have reported a higher prevalence of DM among patients with non-aneurysmal SAH compared to those with aneurysmal SAH, supporting the hypothesis that NASAH may, in part, reflect an underlying microvascular pathology [3,10,11].

A literature review found that non-aneurysmal peri-mesencephalic SAH (NAPMSAH) is more frequently associated with diabetes (17%) compared to aneurysmal SAH, where prevalence typically ranges from 10% to 12% [10]. A study conducted on 4,083 patients with diabetes provides population-based evidence linking T1DM with an increased incidence of NASAH, suggesting a crude SAH incidence of 40.9 per 100,000 person-years among patients with T1DM, substantially higher than rates observed in the general population [4].

Chronic hyperglycemia in diabetes leads to well-established microvascular changes, including endothelial dysfunction, basement membrane thickening, and increased vascular fragility [9,12]. These alterations may predispose small intracranial vessels to spontaneous rupture, even in the absence of aneurysm formation. In this context, NASAH may represent a possible hemorrhagic manifestation of diabetic microangiopathy, analogous to other recognized complications such as retinopathy and nephropathy [4].

In the present case, the patient had a long-standing history of poorly controlled T1DM, as evidenced by significantly elevated HbA1c levels. Importantly, she lacked traditional risk factors for SAH, including hypertension, smoking, trauma, and coagulopathy. Neuroimaging confirmed subarachnoid hemorrhage while excluding aneurysmal or other structural vascular causes through CT angiography. This clinical scenario supports the hypothesis that chronic diabetic microvascular disease may have contributed to vessel fragility and subsequent hemorrhage.

Another important consideration is the pattern and prognosis of NASAH. Peri-mesencephalic and other non-aneurysmal bleeding patterns are often associated with a more favorable outcome [10], as observed in this patient, who experienced clinical improvement without neurological sequelae. Early recognition and appropriate supportive management, including the use of nimodipine and careful monitoring, likely contributed to this favorable course.

This case also highlights important challenges in resource-limited settings. While DSA remains the gold standard for excluding occult vascular lesions [13], it was unavailable at our institution. In such settings, high-quality CT and CT angiography can provide sufficient diagnostic confidence to guide management [13-15]. Clinicians should therefore maintain a high index of suspicion for NASAH in diabetic patients presenting with sudden, severe headache, even when advanced diagnostic tools are limited.

This case has several limitations. First, DSA was not performed because it was unavailable at our institution. Second, although funduscopic examination revealed no evidence of diabetic retinopathy and the patient's serum creatinine level was within the normal range, a comprehensive evaluation for other diabetic microvascular complications, including albuminuria-based assessment of diabetic nephropathy and formal evaluation for peripheral neuropathy, was not performed. Consequently, the proposed contribution of diabetic microangiopathy to the development of NASAH remains hypothetical and should be interpreted with caution.

Overall, this report contributes to the growing body of literature suggesting a potential association between DM and NASAH. While a causal relationship cannot be established from a single case, it underscores the need for further clinical and mechanistic studies to clarify the role of diabetic microangiopathy in intracranial hemorrhagic events.

## Conclusions

This case highlights NASAH as a potential, though uncommon, clinical presentation in patients with long-standing T1DM. In the absence of traditional risk factors and identifiable vascular abnormalities, this case raises the possibility of an association between chronic diabetic vascular changes and NASAH; however, a causal relationship cannot be established from a single case. Clinicians should maintain a high index of suspicion for SAH in diabetic patients presenting with acute severe headache, even in resource-limited settings. Early diagnosis using available imaging modalities and timely supportive management can contribute to favorable clinical outcomes. Further studies are needed to clarify the potential role of diabetic microvascular changes in the pathogenesis of NASAH.

## References

[1] Babiker MS (2024). A case report of a sudden death after recovery from a non-aneurysmal subarachnoid hemorrhage. Trends Med Res.

[2] Adams HP Jr, Putman SF, Kassell NF, Torner JC (1984). Prevalence of diabetes mellitus among patients with subarachnoid hemorrhage. Arch Neurol.

[3] Yuan Y, Chen J, Zhang Y (2022). Exploration of risk factors for poor prognosis of non-traumatic non-aneurysmal subarachnoid hemorrhage. Biomolecules.

[4] Korja M, Thorn LM, Hägg S (2013). Subarachnoid hemorrhage in type 1 diabetes: a prospective cohort study of 4,083 patients with diabetes. Diabetes Care.

[5] (2026). Hunt and Hess grading system (subarachnoid hemorrhage). https://radiopaedia.org/articles/hunt-and-hess-grading-system-subarachnoid-haemorrhage.

[6] (2026). WFNS subarachnoid hemorrhage grading system. https://radiopaedia.org/articles/wfns-subarachnoid-haemorrhage-grading-system.

[7] (2026). Fisher scale of subarachnoid hemorrhage. https://radiopaedia.org/articles/fisher-scale-of-subarachnoid-haemorrhage.

[8] Tarkiainen J, Hovi V, Pyysalo L, Ronkainen A, Frösen J (2023). The clinical course and outcomes of non-aneurysmal subarachnoid hemorrhages in a single-center retrospective study. Acta Neurochir (Wien).

[9] Simpson RK Jr, Contant CF, Fischer DK, Cech DA, Robertson CS, Narayan RK (1991). The influence of diabetes mellitus on outcome from subarachnoid hemorrhage. Diabetes Res.

[10] Roman-Filip I, Morosanu V, Bajko Z, Roman-Filip C, Balasa RI (2023). Non-aneurysmal perimesencephalic subarachnoid hemorrhage: a literature review. Diagnostics (Basel).

[11] Mortazavi ZS, Zandifar A, Ub Kim JD (2023). Re-evaluating risk factors, incidence, and outcome of aneurysmal and non-aneurysmal subarachnoid hemorrhage. World Neurosurg.

[12] Zhang Z, Zhao Y, Liu Y (2022). Effect of stress-induced hyperglycemia after non-traumatic non-aneurysmal subarachnoid hemorrhage on clinical complications and functional outcomes. CNS Neurosci Ther.

[13] Schubert R, Santoso MB, Li Y (2025). Perimesencephalic non-aneurysmal subarachnoid hemorrhage: is there a need for repeat digital subtraction angiography?. Neuroradiology.

[14] Rinkel GJ, Wijdicks EF, Vermeulen M (1991). Nonaneurysmal perimesencephalic subarachnoid hemorrhage: CT and MR patterns that differ from aneurysmal rupture. AJNR Am J Neuroradiol.

[15] Kaim A, Mader I, Kirsch E, Radü EW, Steinbrich W (1995). Perimesencephalic subarachnoid hemorrhage: clinical and computer tomography aspects (Article in German). Rofo.

